# A qPCR and multiplex pyrosequencing assay combined with automated data processing for rapid and unambiguous detection of ESBL-producers *Enterobacteriaceae*

**DOI:** 10.1186/s13568-015-0136-1

**Published:** 2015-08-12

**Authors:** Yann Deccache, Leonid M Irenge, Jérôme Ambroise, Encho Savov, Dan Marinescu, Raphael B Chirimwami, Jean-Luc Gala

**Affiliations:** Defence Laboratories Department, Belgian Armed Forces, Brussels, Belgium; Center for Applied Molecular Technologies, Institut de Recherche Expérimentale et Clinique, Université Catholique de Louvain, B1.30.24, Clos Chapelle-aux-Champs, 1200 Brussels, Belgium; Department of Epidemiology and Hygiene, Military Medical Academy of Sofia, Georgi Sofijsky blvd. 3, 1606 Sofia, Bulgaria; Bucharest Clinical Emergency Hospital, Calea Floreasca 8 Sector 1, Bucharest, Romania; Bukavu General Hospital, Université Catholique de Bukavu, P.O. Box 285, Bukavu, Democratic Republic of Congo

**Keywords:** ESBLs, Detection, qPCR, Multiplex pyrosequencing

## Abstract

Rapid and specific detection of extended-spectrum β-lactamase-producing (ESBL) bacteria is crucial both for timely antibiotic therapy when treating infected patients as well as for appropriate infection control measures aimed at curbing the spread of ESBL-producing isolates. Whereas a variety of phenotypic methods are currently available for ESBL detection, they remain time consuming and sometimes difficult to interpret while being also affected by a lack of sensitivity and specificity. Considering the longer turnaround time (TAT) of susceptibility testing and culture results, DNA-based ESBL identification would be a valuable surrogate for phenotypic-based methods. Putative ESBL-positive *Enterobacteriaceae* isolates (n = 330) from clinical specimen were prospectively collected in Bulgaria, Romania and Democratic Republic of Congo and tested in this study. All isolates were assessed for ESBL-production by the E-test method and those giving undetermined ESBL status were re-tested using the combination disk test. A genotypic assay successively combining qPCR detection of *bla*CTX-M, *bla*TEM and *bla*SHV genes with a multiplex pyrosequencing of *bla*TEM and *bla*SHV genes was developed in order to detect the most common ESBL-associated TEM and SHV single nucleotides polymorphisms, irrespective of their plasmid and/or chromosomal location. This assay was applied on all *Enterobacteriaceae* isolates (n = 330). Phenotypic and genotypic results matched in 324/330 (98.2%). Accordingly, real-time PCR combined with multiplex pyrosequencing appears to be a reliable and easy-to-perform assay with high-throughput identification and fast TAT (~5 h).

## Introduction

Beta-lactams are antimicrobial drugs sharing a β-lactam ring structure (Kong et al. 2009). They are among the most commonly prescribed drugs worldwide, especially for treatment of infections caused by Gram-negative bacteria (GNB) (Poole 2004; Paterson 2006; Pitout et al. 1997). A major shortcoming of this massive use of β-lactams antibiotics is the sharp and steady increase of antimicrobial resistance as evidenced worldwide over the past decades (Pitout and Laupland 2008; Gibold et al. 2014). The main resistance mechanism is the synthesis of β-lactamases which retain the ability of hydrolyzing the β-lactam ring of a wide range of β-lactam antibiotics, including penicillins, cephalosporins and carbapenems (Drawz and Bonomo 2010; Bush 2010). These hydrolyzing enzymes are encoded by genes located either on bacterial chromosomes or on extra-chromosomal transferable mobile elements (Poole 2004). So far, more than 1,300 β-lactamases have been described (Bush 2013) (http://www.lahey.org/studies/) and divided in four molecular classes (A–D) depending on their amino acids sequence (Jacoby and Munoz-Price 2005). Class A β-lactamases, which is mainly found in GNB, is the most prevalent worldwide. Among GNB, extended spectrum β-lactamases (ESBLs) are the most common as illustrated by ESBL-producing *Enterobacteriaceae* which have emerged as an important cause of a wide spectrum of infections (Giamarellou 2005; Owens and Lautenbach 2008). ESBLs confer resistance to penicillins, cephalosporins (first-, second- and third-generation), and aztreonam while retaining susceptibility to clavulanate (Paterson and Bonomo 2005). Many ESBLs are derived from β-lactamase variants arising from amino acids substitutions that enable enzymes to hydrolyze various β-lactam antibiotics (Bradford 2001; Haanperä et al. 2008). Among class A ESBL families, the two main representatives are TEM and SHV in which amino acids substitutions occur in *bla*TEM and *bla*SHV genes (Pfaller and Segreti 2006). In fact, some isolates may carry concomitantly a chromosomal β-lactamase (most commonly SHV-1 or TEM-1) and the plasmid variants derived from the chromosomal *bla*SHV or *bla*TEM gene. This chromosomal β-lactamase may interfere with molecular detection of the ESBL gene usually residing in plasmids (Haanperä et al. 2008; Jones et al. 2009).

Another major ESBL family is CTX-M which derives from *Klyuvera* spp (Rossolini et al. 2008). The successful spread of ESBLs in a wide range of *Enterobacteriaceae* can be attributed to the fact that the genes coding for ESBLs are often located on self-transmissible or mobilizable broad-host-range plasmids (Livermore et al. 2007; D’Andrea et al. 2013).

Extended-spectrum β-lactamase detection is crucial both for infection control measures as well as for selecting appropriate antimicrobial therapy, as failure to rapidly and unambiguously identify ESBL-producing isolates may delay the initiation of appropriate infection control measures and further contribute to their spread in hospital and community settings (Bradford 2001).

In the majority of healthcare facilities, the routine detection of ESBL production by *Enterobacteriaceae* is actually carried out by phenotypic methods (Pitout and Laupland 2008). These tests are based on the principle that most ESBLs hydrolyze third-generation cephalosporins and are inhibited by clavulanic acid (Robin et al. 2008; Jarlier et al. 1988; Cormican et al. 1996; Carter et al. 2000). Most guidelines recommend to first screen isolates for decreased susceptibility to extended-spectrum cephalosporins in primary susceptibility testing, and then to carry out a confirmatory test to verify ESBL production (Gazin et al. 2012; Garrec et al. 2011; Rupp and Fey 2003). However, and despite the variety of phenotypic methods available and the existence of various guidelines available for the phenotypic detection of ESBL-producing bacteria, this remains a contentious issue, and proficiency testing shows that compliance varies widely across different parts of the world (Pitout and Laupland 2008). For instance, it has been reported that laboratories fail to interpret correctly the inhibition ellipse in 30% of cases. Indeed, the enzymes may vary in their substrate affinities and in their catalytic efficiencies, and also differ in their penetration rates into bacterial cells (Drieux et al. 2008). Furthermore, the emergence of AmpC β-lactamases complicates the detection of ESBL-production. Indeed, AmpC β-lactames display high resistance rate to cephalosporins without retaining any susceptibility to clavulanate (Polsfuss et al. 2011). This observation is important, considering the recent emergence of plasmidic AmpC β-lactamases species like *Escherichia coli* and *Klebsiella* spp (Jacoby 2009).

In that respect, DNA-based detection of ESBL-production appears as a valuable alternative to the phenotypic-based methods. It is indeed independent of gene expression and relatively rapid as compared with susceptibility testing and culture results. In addition, this type of assay could help unravel issues related to ESBL-producing isolates, among which false-negative or indeterminate results.

Several molecular tests aiming to detect and/or characterize TEM, SHV and CTX-M have previously been described (Naas et al. 2007; Haanperä et al. 2008; Oxacelay et al. 2009; Jones et al. 2009; Leinberger et al. 2010; Cohen Stuart et al. 2010; Al-Agamy et al. 2014). However, despite these encouraging achievements, technological limitations still hamper their implementation in routine microbiology practice, mainly due to the fact that they are still cumbersome and time-consuming (Drieux et al. 2008; Wintermans et al. 2013). Consequently, there is still a need for molecular high-throughput techniques detecting rapidly and reliably ESBL-producing isolates.

The aim of the current study was to develop and test a multiplex pyrosequencing assay for quick and unambiguous detection of ESBL-producing *Enterobacteriaceae* isolates. In this work, real-time PCR amplification of *bla*CTX-M, *bla*SHV and *bla*TEM genes was completed by multiplex pyrosequencing characterization of *bla*TEM and *bla*SHV in order to detect ESBL associated variants in isolates from Bulgaria, Romania and Democratic Republic of Congo.

## Materials and methods

### Bacterial isolates

Various clinical specimens [including blood culture, peritoneal, pleural and pericardial fluids, urine, pus and sputum; n = 330)] were collected between 2008 and 2014 from patients from Bucharest Clinical Emergency Hospital (Romania; n = 177), from the Intensive Care Unit (ICU) at the Military Medical Academy of Sofia (Bulgaria; n = 144) and from various hospital wards of the Bukavu Provincial General Hospital (South Kivu province, Democratic Republic of Congo; n = 9). After initial culture, presumptive *Enterobacteriaceae* isolates from DR Congo were identified using standard microbiological methods whereas isolates from Bulgaria and Romania were identified using the Vitek-2 automated instrument ID system (BioMérieux, Marcy l’Etoile, France). Four commercial strains were used as negative and positive controls: ATCC-35218 (non-ESBL producing *E. coli*), ATCC-700603 (SHV-18 type β-lactamase producing *K. pneumoniae*), DSM-22314 (TEM-46 ESBL producing *E. coli*), and DSM-22313 (TEM-50 ESBL producing *E. coli*), (see Table 1).

**Table 1:** Commercial reference strains used as negative and positive controls

	TEM_104_	TEM_164_	TEM_238_	SHV_179_	SHV_238_	SHV_240_	Phenotype
ATCC-35218 (*E. coli*)	Glu_104_	Arg_164_	Gly_238_	–	–	–	Non-ESBL
ATCC-700603 (*K. pneumoniae*)	–	–	–	Asp_179_	Gly_238_Ala	Glu_240_Lys	SHV-18
DSM-22313 (*E. coli*)	Glu_104_Lys	Arg_164_	Gly_238_Ser	–	–	–	TEM-46
DSM-22314 (*E. coli*)	Glu_104_Lys	Arg_164_Ser	Gly_238_	–	–	–	TEM-50

### Susceptibility testing

In Romania and Bulgaria, ESBL-production assay was performed using Vitek-2 cards whereas in DRC, it was carried out by the double-disk synergy test on Mueller–Hinton II^®^ Agar (Oxoid Ltd, Cambridge, UK) using disks soaked with ceftazidime or cefotaxime (30 µg each) placed at a distance of 20 mm apart from a disk containing amoxicillin plus clavulanic acid (10 µg). A clear-cut enhancement of the inhibition in front of either ceftazidime and cefotaxime disks towards the clavulanic acid-containing disk (also called “champagne-cork” or “keyhole”) was interpreted as positive for ESBL production (Drieux et al. 2008).

E-test^®^ strips (BioMérieux, Marcy l’Etoile, France) were used for confirmation of ESBL-production. Minimum inhibitory concentrations (MIC) of ceftazidime and cefotaxime with and without clavulanic acid were determined after 16–18 h incubation on ISO Sensitest^®^ Agar plates inoculated with suspension of isolates at a fixed density (0.5–0.6 McFarland standard). The test was performed and interpreted according to the manufacturer’s instructions. *Escherichia coli* ATCC 35218 and *Klebsiella pneumoniae* ATCC 700603 strains were used as ESBL negative and positive controls, respectively.

Each isolate generating a non-determinate (ND) result with this gradient test was retested using the combination disk test (CDT) as described by Carter (Carter et al. 2000). In brief, disks containing 30 µg of cefotaxime, ceftazidime or cefepime and disks containing a combination of each of these drugs with 10 µg clavulanic acid (Bio-Rad, Nazareth Eke, Belgium) were placed independently on a 0.5 McFarland opacity lawn culture of the tested isolate on a ISO Sensitest^®^ Agar plate and incubated for 18–24 h at 37°C. Isolates were considered ESBL-producing if the inhibition zone measured around one of the combination disks was at least 5 mm larger that of the corresponding cephalosporin disk.

### DNA extraction and DNA-based identification of isolates

For each isolate, a single colony was selected after overnight growth on ISO-Sensitest^®^ agar plate and cultured in fresh ISO-Sensitest^®^ broth (Oxoid Ltd, Cambridge, UK) for 6 h. The broth was centrifuged and DNA subsequently extracted from the pelleted cells using a DNA tissue kit with on the BioRobot EZ1 (Qiagen, Hilden, Germany) according to the manufacturer’s instructions.

Purified DNA was quantified using NanoDrop ND1000^®^ spectrophotometer (Nanodrop Technologies, Inc. Wilmington, DE, US) and stored at −20°C for further molecular assays. In case of ambiguous species identification with the Vitek-2 system, DNA-based identification was then performed as described previously (Bosshard et al. 2006; Vandercam et al. 2008).

### Polymerase chain reaction (PCR) amplification and pyrosequencing assay

#### Primers design

All publicly available nucleotide sequences of *bla*TEM and *bla*SHV alleles related to ESBL-production phenotype (http://www.lahey.org/Studies/) were retrieved from the GenBank nucleotide sequence database and aligned using the BioNumerics software v7.1. (Applied Maths, Sint-Martens-Latem, Belgium). A total of 88 *bla*TEM and 30 *bla*SHV nucleotide sequences were aligned. Consequently, two simplex qPCRs were designed to amplify a 302 bp sequence flanking amino acids 179, 238 and 240 of *bla*SHV and a 500 bp sequence flanking positions 104, 164 and 238 of *bla*TEM (Table 2). The aforementioned positions include indeed the most frequent substitutions associated with ESBL-production phenotype (Cohen Stuart et al. 2010). A duplex qPCR was used to amplify two *bla*CTX-M fragments of 224 and 175 bp as previously described (Naas et al. 2007).

**Table 2:** List of primers used for ESBL-encoding gene detection and pyrosequencing in *Enterobacteriaceae*

Target gene	Primer name	Sequence 5′–3′	qPCR	Pyrosequencing assay	Reference
*bla*TEM	TEM-Forward	biotin-CGCCGCATACACTATTCTCA	Simplex	Triplex	Own primer design
	TEM-Reverse	ATACGGGAGGGCTTACCATC			
	TEM-seq104	TGCTTTTCTGTGACTGGTG			
	TEM-seq164	TTCAGCTCCGGTTCC			
	TEM-seq238	CGCKAGAHCCACGCT			
*bla*SHV	SHV-Forward	biotin-TCTGYGCCGCCGTCATTA	Simplex	Duplex	Own primer design
	SHV-Reverse	CTTTGTTATTCGGGCCAAGCA			
	SVH-seq179	GCTGGCCGGGGATGTG			
	SVH-seq238	ATSCCGCGCSCACCCC			
*bla*CTX-M	CTX-1Forward	ATGTGCAGYACCAGTAARGTGA	Duplex	–	Naas et al. (2007)
	CTX-1Reverse	TGRGMAATCARYTTRTTCAT			
	CTX-2Forward	TGGTRAYRTGGMTBAARGG			
	CTX-2Reverse	TGGGTRAARTARGTSACCAGAA			

Degenerate base symbols (IUPAC code): K represents G/T, Y is T/C, S is G/C, R is G/A, M is A/C, H is A/T/C and B is G/T/C.

#### Simplex and duplex qPCR amplification

Each qPCR was carried out in 50 μL of a reaction mixture containing 100 pg of extracted DNA as template, 300 nM of each primer, and 25 μL of Power SYBR^®^ Green Reagents 2x (Applied Biosystems, Nieuwekerk, The Netherlands). Amplifications were performed either on a 7900HT Fast Real-Time PCR System (Applied Biosystems, Nieuwekerk, The Netherlands) or a Stratagene Mx3005P (Agilent Technologies, Santa Clara, CA, USA). The reaction was initiated at 50°C for 2 min and 95°C for 10 min followed by 45 cycles at 95°C for 15 s and 63°C for 1 min for the TEM and SHV qPCRs. The duplex CTX-M was carried out using the same conditions as above except that the optimal annealing temperature was 57°C. Finally, a melting curve analysis was performed. Data were analyzed using the analytical software SDS 2.4 from Applied BioSystem or MxPro qPCR software from Agilent Technologies.

#### Multiplex pyrosequencing protocol

Pyrosequencing is a bioluminometric assay allowing rapid and high throughput sequencing of short DNA sequences. This type of assay has already proved it value for assessing drug-resistance determining regions in various bacteria (Deccache et al. 2011; Haanperä et al. 2005). In order to increase the throughput of the assay, two multiplex pyrosequencing assays were concomitantly developed using three and two pyrosequencing primers (Table 2) to generate corresponding sequences for *bla*TEM and *bla*SHV amplicons, respectively.

The pyrosequencing assay was performed using a PyroGold SQA sample preparation kit (Qiagen, Hilden, Germany) according to manufacturer’s instructions. Briefly, 40 µl of biotinylated PCR products were immobilized for 15 min using 4 µl streptavidin-coated Sepharose beads (GE Healthcare-Biosciences AB, Uppsala, Sweden) and captured using the Vacuum Prep Tool (Qiagen, Hilden, Germany) according to manufacturer’s instructions. After a denaturation step (NaOH 0.5 M during 5 s) and a wash step for the removal of unlabeled complementary DNA of the amplicon, the biotinylated single-stranded DNA present on the streptavidin-coated Sepharose beads were transferred to a 96-well plate and used as template for the pyrosequencing assay with a mixture of sequencing primers. The TEM mix contained 6, 20 and 60 pmol of the TEM-seq104, TEM-seq164 and TEM-seq238 primers, respectively, whilst the SHV mix contained 60 and 20 pmol of the SHV-seq179 and SHV-seq238 primers, respectively. The pyrosequencing nucleotide dispensation orders were selected using SENATOR (SElecting the Nucleotide dispensATion Order) algorithm (Ambroise et al. 2013). Nucleotide dispensation orders with 15 (CAGCCTGACATATCA) and 11 (GTGACTGCGTC) nucleotides were selected for the triplex TEM and duplex SHV assays, respectively. Pyrosequencing was carried out using a PSQ 96 MA system (Qiagen, Hilden, Germany).

All multiplex pyrosequencing signals generated for TEM and SHV were analyzed using a new web interactive application (available at https://ucl-irec-ctma.shinyapps.io/Pyrosequencing-TEM-SHV and in ‘download’ section of http://www.uclouvain.be/ctma.html where a template data set can be downloaded) written with R.3.1.2 software (Ihaka and Gentleman 1996) using the “shiny” package. This new application was developed using an improved version of three previously published algorithms including (1) AdvISER-PYRO for analyzing low and complex signals resulting from samples including several mycobacteria (Ambroise et al. 2013), (2) AdvISER-M-PYRO for analyzing overlapping pyro-signals generated from multiplex reactions conducted on mono-allelic genes in bacteria (Ambroise et al. 2014), and (3) AdvISER-MH-PYRO for analyzing overlapping pyro-signals generated from multiplex reactions to genotype bi-allelic human SNP (Ambroise et al. 2015). While the initial version of AdvISER-M-PYRO analyzed multiplex pyrosequencing signals by selecting a single and unique sequence for each genomic region, this new version enables the analysis of multiplex pyrosequencing signals generated from samples including two distinct sequences (i.e., plasmid and chromosomal) for each genomic region.

**Fig. 1:**
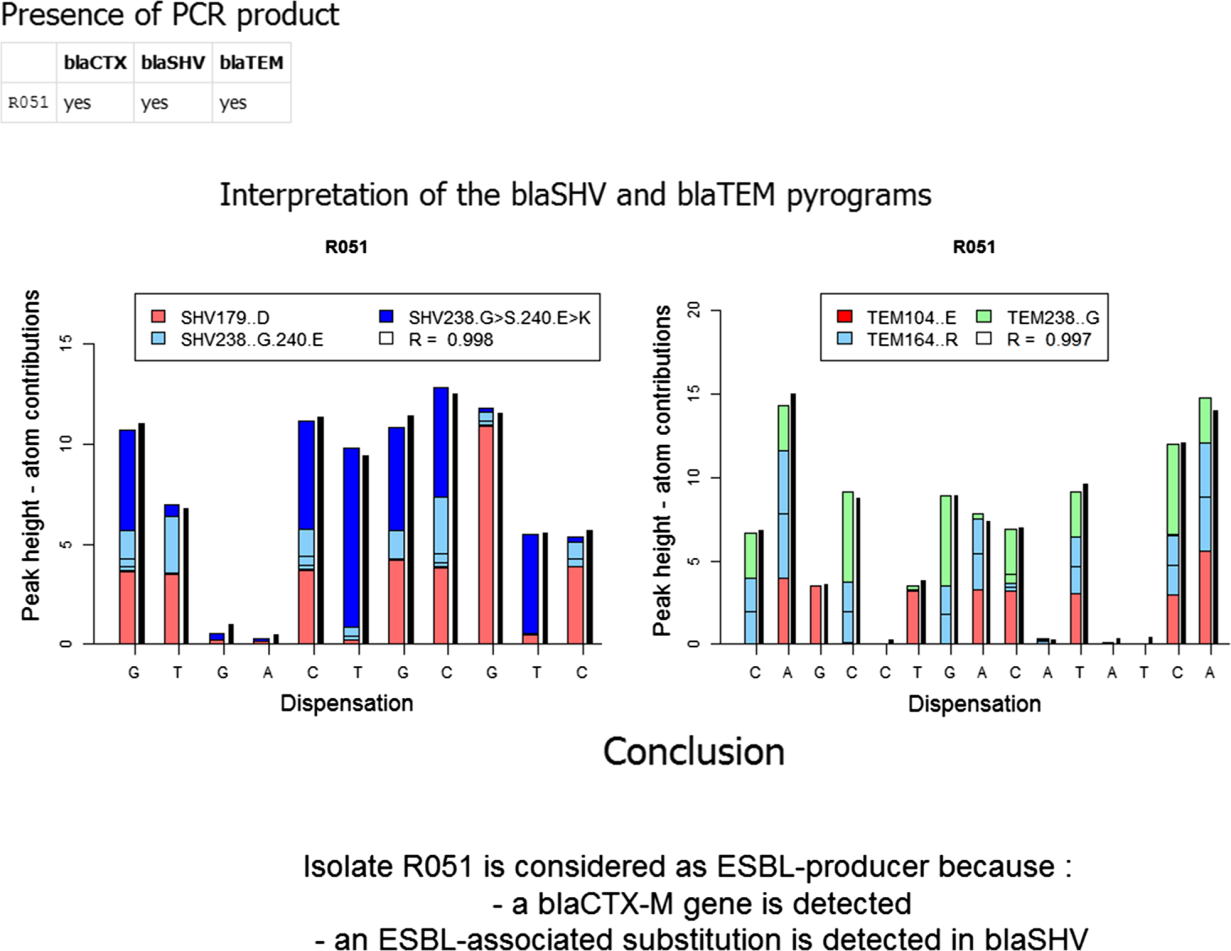
Output illustration of the web interactive application. For a given isolate, integration of PCR and multiplex pyrosequencing results allows the determination of the ESBL-producing status (available at https://ucl-irec-ctma.shinyapps.io/Pyrosequencing-TEM-SHV/). As demonstrated in this example, the algorithm enables to correctly decompose the multiplex pyrosequencing signal generated for the *bla*SHV gene despite the presence of two sequences for the *bla*SHV238-240 genomic region (i.e., G238 and E240 on one variant and the G238S-E240K double substitutions on another one).

The new web interactive application enables also the integration of PCR and pyrosequencing results in order to correctly identify the ESBL status of each isolate. In that respect, an isolate was considered as ESBL-producer organism if either one or more of the ESBL-associated substitutions was detected from pyrosequencing signals of *bla*TEM or *bla*SHV, or if the *bla*CTX-M gene was present (as illustrated on Fig. 1). On the contrary, the isolate was considered as non-ESBL-producer organism when *bla*TEM, *bla*SHV and *bla*CTX-M genes were either not detected or when amplified *bla*SHV or *bla*TEM genes did not carry any ESBL-associated substitution. Pyrosequencing signals displaying low signal-to-noise ratio (maximum peak height <10 relative fluorescence units) and/or a low confidence index (R < 0.99) were considered as unsafe and re-tested using the same protocol.

### Sequencing analysis of amplicons

Sanger sequencing was used to confirm pyrosequencing results. Amplifications products of *bla*TEM and *bla*SHV were purified using MSB^®^ Spin PCRapace purification kit (STRATEC Molecular GmbH, Berlin, Germany) according to manufacturer’s instructions. After having assessed 5 µl of each purified PCR product on a 2% (w/v) agarose gel stained with ethidium bromide, corresponding amplicons were sequenced in both directions on an automated ABI 3130 Genetic Analyser apparatus (Applied Biosystems, Nieuwekerk, The Netherlands), using the BigDye Terminator v3.1 Cycle Sequencing kit from the same manufacturer. Nucleotide and deduced amino acid sequences were analyzed using the BioNumerics software v7.1. (Applied Maths, Sint-Martens-Latem, Belgium) and compared to publically available database.

## Results

Phenotypic identification of all isolates (n = 330) was as follows: *Klebsiella pneumonia* (n = 147), *Escherichia* coli (n = 119), *Klebsiella* spp. (n = 30)*, Enterobacter cloacae* (n = 16)*, Proteus mirabilis* (n = 10), *Serratia marcescens* (n = 6), and *Morganella morgannii* (n = 2).

### Phenotypic results

ESBL- and non-ESBL-producing organisms were assessed by E-test assay in 267/330 (80.9%) and 20/330 (6.1%) isolates, respectively whereas 43/330 (13%) presented a MIC value lower than the predefined range, hence yielding an ND result. CDT was used to retest these 43 isolates displaying a ND E-test, whereupon 36 isolates were found ESBL-producers and 7 non-ESBL producers.

### Genotypic results

The newly-developed multiplex pyrosequencing assay was used to rapidly assess the ESBL-producing status of all isolates following *bla*TEM, *bla*SHV and *bla*CTX-M amplification by qPCR. Among all isolates (n = 330), 295 (89.4%) produced high-quality pyrosequencing signals (maximum peak height >10, R > 0.99) while 35 (10.6%) isolates did not meet these quality criteria and where therefore re-tested with the same protocol. After this second round of analysis, 308 (93.3%) and 22 (6.7%) isolates were identified as ESBL- and non-ESBL-producing bacteria, respectively.

The TAT for the analysis was ~5 h (i.e., 40 min for DNA purification, 1h50 for the duplex qPCR *bla*CTX-M, 1h50 for the concomitant simplex TEM and SHV qPCR, and 40 min for the multiplex pyrosequencing and data processing using the new web interactive application).

It is of note that the Sanger sequence analysis of all *bla*SHV and *bla*TEM amplification fragments was perfectly concordant with the pyrosequencing results (data not shown).

### Phenotypic: genotypic comparison

Comparison between DNA-based and E-test results is detailed in Table 3. When CDT test was combined with E-test, a quasi-perfect concordance with results from the multiplex DNA-assay was observed (Table 4). Altogether after CDT retesting, six results remained discordant: one *P. mirabilis* and four *E. coli* isolates considered as ‘non-ESBL producing bacteria’ by CDT despite carrying the *bla*CTX-M gene (Table 4). Conversely, R080 isolate was considered as ‘ESBL-producing bacteria’ according to E-test, while neither *bla*SHV, *bla*TEM, nor *bla*CTX-M genes were detected by qPCR. The ESBL phenotype was however confirmed after re-testing this isolate with CDT.

**Table 3:** Distribution of the 330 isolates tested, depending on the species, phenotypic test (E-test) and DNA-based test

	E-test	DNA-based test				n
		CTX-M	TEM	SHV	Interpretation	
*E. cloacae*, n = 16	ESBL	Absent	Wild-type	Mutated	ESBL	1
		Present	Absent	Absent	ESBL	1
			Wild-type	Absent	ESBL	8
	ND	Absent	Wild-type	Mutated	ESBL	1
		Present	Wild-type	Absent	ESBL	4
	Non-ESBL	Absent	Wild-type	Mutated	ESBL*	1*
*E. coli*, n = 119	ESBL	Present	Absent	Absent	ESBL	34
			Wild-type	Absent	ESBL	50
				Mutated	ESBL	2
	ND	Present	Wild-type	Absent	ESBL	20
	Non-ESBL	Absent	Absent	Absent	Non-ESBL	3
		Absent	Wild-type	Absent	Non-ESBL	10
*K. pneumoniae*, n = 147	ESBL	Absent	Absent	Absent	Non-ESBL*	1*
			Wild-type	Mutated	ESBL	8
		Present	Absent	Wild-type	ESBL	4
			Wild-type	Absent	ESBL	4
				Wild-type	ESBL	73
				Mutated	ESBL	41
	ND	Absent	Absent	Absent	Non-ESBL	2
			Wild-type	Mutated	ESBL	3
		Present	Wild-type	Absent	ESBL	1
				Wild-type	ESBL	5
				Mutated	ESBL	1
	Non-ESBL	Absent	Absent	Absent	Non-ESBL	2
				Wild-type	Non-ESBL	1
			Wild-type	Wild-type	Non-ESBL	1
*Klebsiella* spp., n = 30	ESBL	Present	Absent	Wild-type	ESBL	1
			Wild-type	Absent	ESBL	13
				Wild-type	ESBL	5
				Mutated	ESBL	10
	ND	Present	Wild-type	Absent	ESBL	1
*P. mirabilis*, n = 10	ESBL	Present	Wild-type	Absent	ESBL	1
				Wild-type	ESBL	1
				Mutated	ESBL	2
	ND	Absent	Wild-type	Mutated	ESBL	3
		Present	Absent	Absent	ESBL	1
	Non-ESBL	Absent	Absent	Absent	Non-ESBL	2
*S. marcescens*, n = 6	ESBL	Present	Wild-type	Absent	ESBL	5
	ND	Present	Wild-type	Absent	ESBL	1
*M. morganii*, n = 2	ESBL	Present	Absent	Absent	ESBL	1
			Wild-type	Absent	ESBL	1

* Isolates for which genotypic and phenotypic results are discordant. Both discordant isolates and isolates for which E-test yielded a ‘ND’ result (n = 43) were re-tested using the combination disk test (CDT).

**Table 4:** Distribution of the 45 isolates re-tested using the CDT (43 with a ND and 2 with discrepant results between E-test and DNA-based assay)

	Phenotypic test			DNA-based test			n
	E-test	CD	CTX-M	TEM	SHV	Interpretation	
*E. cloacae*, n = 6	ND	ESBL	Absent	Wild-type	Mutated	ESBL	1
		ESBL	Present	Wild-type	Absent	ESBL	4
	Non-ESBL (MMA55)*	ESBL*	Absent	Wild-type	Mutated	ESBL*	1*
*E. coli*, n = 20	ND	ESBL	Present	Wild-type	Absent	ESBL	16
		Non-ESBL*	Present	Wild-type	Absent	ESBL*	4*
*K. pneumoniae*, n = 13	ND	Non-ESBL	Absent	Absent	Absent	Non-ESBL	2
		ESBL	Absent	Wild-type	Mutated	ESBL	3
			Present	Wild-type	Absent	ESBL	1
					Wild-type	ESBL	5
					Mutated	ESBL	1
	ESBL (R080)*	ESBL*	Absent	Absent	Absent	Non-ESBL*	1*
*Klebsiella* spp., n = 1	ND	ESBL	Present	Wild-type	Absent	ESBL	1
*P. mirabilis*, n = 4	ND	ESBL	Absent	Wild-type	Mutated	ESBL	3
		Non-ESBL*	Present	Absent	Absent	ESBL*	1*
*S. marcescens*, n = 1	ND	ESBL	Present	Wild-type	Absent	ESBL	1

* Isolates for which genotypic and phenotypic results are discordant. Both discordant isolates and isolates for which E-test yielded a ‘ND’ result (n = 43) were re-tested using the combination disk test (CDT).

It is of note that another discordant result was observed between E-test and DNA-based test on *E. cloacae* MMA55 isolate. This isolate was characterized as ‘non-ESBL producing bacteria according to the E-test, despite the presence of a *bla*SHV gene with point mutations leading to a double E_240_K and G_238_S ESBL-associated substitution. CDT confirmed however the status of ESBL-produced. The isolate should therefore not be considered as a phenotypic-genotypic discordant.

## Discussion

A new DNA-based assay has been developed for enabling rapid and unambiguous detection of ESBL-producing *Enterobacteriaceae* isolates. The method consists of a qPCR amplification of *bla*TEM, *bla*SHV and *bla*CTX-M and subsequent multiplex pyrosequencing of ESBL production-related mutations within *bla*SHV and *bla*TEM genes. All selected mutations have previously been reported (Naas et al. 2007; Haanperä et al. 2008; Oxacelay et al. 2009; Jones et al. 2009; Leinberger et al. 2010; Cohen Stuart et al. 2010; Al-Agamy et al. 2014). Rapid and unambiguous detection methods for ESBL-production are paramount for appropriate management of infectious diseases as it has long been recognized that prompt and targeted antibiotic therapy improves the outcome for patients with infectious diseases (Perez et al. 2007). Furthermore, rapid laboratory detection is critical for the development of infection control measures aimed at reducing the transmission rate of antibiotic-resistant microorganisms in hospital wards (Stürenburg and Mack 2003). Up to now, phenotypic methods have routinely been used for detecting antimicrobial resistant isolates, and particularly ESBL-producing isolates (Robin et al. 2008; Jarlier et al. 1988; Cormican et al. 1996; Carter et al. 2000). Unfortunately, recent studies have consistently pinpointed the many drawbacks affecting these methods. These include a long TAT (24–72 h) and somehow low sensitivity and specificity. As an illustration, Garrec and co-workers (Garrec et al. 2011) compared various phenotypic methods for detecting ESBL production in *Enterobacteriaceae* and found the sensitivity and specificity of ESBL-detection using the Vitek-2 to vary between 92 and 95% in ESBL-producing *E. coli* isolates while being substantially lower (i.e., 50–79%) in ESBL-producing *K. pneumonia* isolates. Lower sensitivity (71%) and specificity (73%) were also reported when using the E-test for detecting ESBL-production in *Enterobacteriaceae* isolates (Garrec et al. 2011). Moreover, E-test method often yields ND results with some isolates (Leverstein-van Hall et al. 2002). Finally, interpretation of results according to various breakpoints also impacts the interpretation of ESBL-production.

Multiplex DNA-based assays targeting specific mutations related to ESBL-production phenotype are expected to circumvent at least part of these difficulties. Whereas molecular methods are usually considered as exquisitely sensitive and specific for rapidly and directly assessing the ESBL-production status in bacterial isolates, it should be pointed out that few of them have, up to now, convincingly demonstrated their capacity to be usefully translated into the routine practice of clinical microbiology. Consequently, the main goal was to develop a multiplex DNA-based assay which could easily be implemented in a clinical setting, while swiftly delivering a reliable ESBL-status for *Enterobacteriaceae* isolates. In that respect, qPCR amplification, multiplex pyrosequencing, and signal analysis were combined in order to develop a quick and reliable assay. The signal analysis algorithm was implemented in a new web interactive application that allowed to correctly decomposing triplex and duplex pyrosequencing signals. While a concomitant presence of chromosomal and plasmid β-lactamase variants within the same isolate does not preclude determining its ESBL phenotype, it may affect the pyrosequencing result of ESBL genes located on the plasmid (Haanperä et al. 2008; Jones et al. 2009). Consequently, the new algorithm was designed to correctly deciphering multiplex pyrosequencing signals generated from samples including two distinct sequences (i.e., plasmid and chromosomal) for each genomic region, as illustrated in Fig. 1.

Compared to phenotypic testing (E-test or CDT), the TAT for the combined multiplex assay was short (~5 h). In addition, result interpretation is rendered easier and more reproducible by the use of the new web interactive application. To further shorten the TAT, it should also be noted that DNA extraction could be sidestepped while carrying out multiplex amplification directly in triplicate with each isolated bacterial colony, after boiling. Moreover, this multiplex assay appears suitable for simultaneous and high-throughput determination of ESBL-production status on up to 30 isolates per run. Conversely, only 6 isolates could be processed per run if the same test was to be carried out in conventional simplex pyrosequencing format (i.e., one well per pyrosequencing reaction). While the difference between low-cost phenotypic test and DNA-based protocol was important when considering conventional simplex pyrosequencing, the combination of the five uniplex pyrosequencing reactions into two multiplex reactions allowed reducing this gap.

In this study, 43 isolates did not yield MIC values compatible with ESBL-production when using E-test strips. This prompted us to use CDT as complementary test in order to assess and confirm the ESBL-status. As indicated above, this process results in extending the TAT which, in turn, could be detrimental for the patient’s health. With the latter 43 isolates, the multiplex DNA-based assay gave rapid and unambiguous ESBL characterization. Comparing the results of genotypic and phenotypic tests showed however a discrepant result with five isolates (i.e., four *E. coli* and one *P. mirabilis*). In all discrepant cases, E-test and CDT gave an indeterminate result and ‘non-ESBL’ phenotype, respectively. Considering the presence the *bla*CTX-M gene in the five isolates, it seems clear that CDT produced false-negative results. According to guidelines published by the European Committee on Antimicrobial Susceptibility Testing (Giske et al. 2013), this type of results may occur when bacteria expressed either a high-level AmpC β-lactamase (Drieux et al. 2008; Jacoby 2009; Munier et al. 2010), either carbapenemases (such as KPCs or MBLs Tsakris et al. 2009; March et al. 2010) and/or severe permeability defects. The presence of such mechanisms could mask the presence of ESBLs.

The case of MMA55 (*E. cloacae*) isolate is also interesting as it pinpoints the added value of the current multiplex DNA-based assay compared to phenotypic tests. This isolate was indeed initially characterized as ‘non-ESBL’ by the gradient test even though multiplex qPCR and pyrosequencing revealed the presence of a SHV variant harboring ESBL-associated substitutions (E_240_K and G_238_S). Subsequent CDT results were however in line with the ESBL-genotypic result.

Another interesting case was R080 (*K. pneumoniae*) isolate. Though clearly displaying an ESBL-phenotype, this isolate was lacking the three antibiotic resistance determinant genes as targeted by our qPCR assay. This phenotype could result from the presence of less frequent ESBL genes, such as PER, VEB, SME or BEL. Isolates displaying a similar pattern have indeed previously been described by others (Nijhuis et al. 2012). Investigating these rare genes fell however beyond the immediate scope of this work but investigations will be pursued to understand this apparent discrepancy and to test this hypothesis. By the way, the flexibility of this multiplex pyrosequencing assay makes it perfectly suitable for adding new ESBL family or genetic variant pending their discovery and demonstration of interest.

Like with most DNA-based tests, it must be emphasized that the current assay detects a spectrum of common and highly prevalent ESBL-associated mutations without a precise identification thereof. If this information is required, full-length gene sequencing would need to be carried out for characterization of the genetic variant for point mutations.

One major potential drawback which so far precludes implementing DNA-based methods in routine microbiology is the risk of carry-over contamination. Whereas this risk needs always to be thoroughly assessed, it can be lowered to an acceptable threshold by incorporating UNG-ampErase in PCR mix (Longo et al. 1990) as well as by performing qPCR preparations and pyrosequencing reactions in separate rooms.

In conclusion, the assay developed in this study can be considered as a *bona fide* surrogate for ESBL-detection in clinical settings. Moreover, the current multiplex pyrosequencing assay is an innovative high-throughput; rapid and reliable assay drastically limiting the use of DNA while reducing analytical costs and waste material disposal.
